# Hemodynamic instability and retinal vein occlusion in glaucoma: Comparative analysis of heart rate variability and choroidal perfusion

**DOI:** 10.1371/journal.pone.0324110

**Published:** 2026-03-06

**Authors:** Ji Hye Lee, Young-Hoon Park

**Affiliations:** Department of Ophthalmology and Visual Science, Seoul St. Mary’s Hospital, College of Medicine, The Catholic University of Korea, Seoul, Republic of Korea; Icahn School of Medicine at Mount Sinai, UNITED STATES OF AMERICA

## Abstract

**Purpose:**

To assess the hemodynamic and structural differences between glaucoma patients who developed retinal vein occlusion (RVO) and those who did not.

**Study design:**

Retrospective, single-center, case-control study

**Methods:**

This study included glaucoma patients who underwent a heart rate variability (HRV) test between January 2018 and July 2024. Patients were subdivided into RVO and non-RVO groups. Baseline mean deviation (MD) and pattern deviation (PSD) of the visual field and optical coherence tomography parameters were analyzed.

**Results:**

Twenty-nine glaucoma patients with RVO and 34 glaucoma patients without RVO were included. Baseline MD and PSD had no difference (MD: −5.98 ± 9.05 vs. −3.70 ± 4.70, *p* = 0.114; PSD: 3.88 ± 3.78 vs. 3.98 ± 3.96, *p* = 0.883). HRV parameters, specifically Standard Deviation of NN interval (SDNN) and root-mean-square of successive differences (rMSSD), were significantly lower in the RVO group (SDNN: 22.12 ± 8.27 vs. 36.71 ± 24.74, *p* = 0.002; rMSSD: 16.34 ± 9.55 vs. 29.87 ± 31.58, *p* = 0.022). A significant difference in choroidal vascularity index was also observed between groups (64.62 ± 7.38 vs. 67.49 ± 5.90, *p* = 0.045). In a parsimonious multivariable logistic regression model, no variable retained statistical significance.

**Conclusion:**

This study suggests that reduced heart rate variability, reflecting autonomic dysfunction, is associated with the development of RVO in glaucoma patients. Although these associations did not remain statistically significant in multivariable analysis, the consistent univariate findings indicate that HRV may serve as a potential vulnerability marker rather than an independent predictor. Further prospective studies are warranted to clarify the clinical utility of HRV in risk stratification for RVO in glaucoma patients.

## Introduction

Despite the development of many treatment options, such as intravitreal anti-vascular endothelial growth factor (anti-VEGF) agents, steroid injection, and laser treatment, retinal vein occlusion (RVO) remains the second most common retinal vascular disease after diabetic retinopathy, and is a leading cause of severe vision loss [1,2]. RVO can be classified based on the site of occlusion: branch RVO occurs mainly at an arteriovenous intersection, and central RVO occurs at or near the lamina cribrosa of the optic disc [1,3]. RVO is associated with various systemic risk factors, including advanced age, hypertension, diabetes mellitus, dyslipidemia, atherosclerosis, and blood viscosity [4–7]. In addition to these systemic risk factors, glaucoma is the primary ocular risk factor for RVO [6,8–10]. Shin et al. [11] reported that autonomic dysfunction can cause blood flow abnormalities in glaucoma patients, potentially accelerating disease progression. This hemodynamic instability could contribute to both the progression of glaucoma and the development of RVO, as RVO is closely related to systemic vascular health.

This study aims to identify the risk factors in glaucoma patients who are more likely to develop RVO and investigate whether autonomic dysfunction in glaucoma patients could increase the risk of RVO development.

## Methods

### Ethics statement

This retrospective, single-center case-control study was approved by the Institutional Review Board (IRB) of Seoul St. Mary’s Hospital, Catholic University of Korea (KC24RASI0497), and adhered to the tenets of the Declaration of Helsinki. As the study was conducted retrospectively, the local IRB of the Catholic University of Korea waived the need for informed consent. Data were accessed between 11/10/2024 and 20/10/2024, following IRB approval.

### Participants

**Fig 1:**
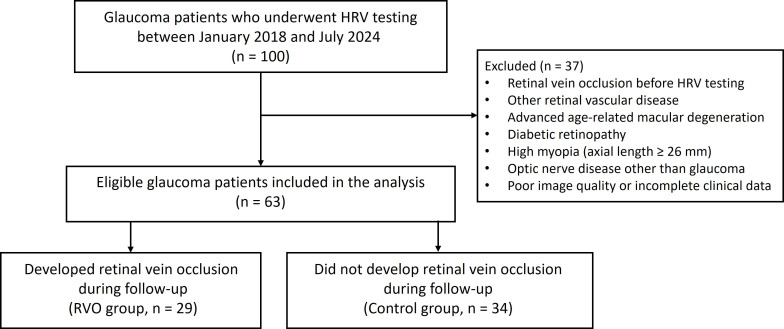
Patient Selection Flowchart. The final study population consisted of 63 eligible glaucoma patients, including 29 who developed retinal vein occlusion during follow-up.

We retrospectively reviewed the medical records of glaucoma patients who underwent heart rate variability (HRV) testing between January 2018 and July 2024 at a single tertiary referral center. HRV was assessed at baseline during routine glaucoma follow-up prior to the development of retinal vein occlusion. Patients were subdivided into two groups: those who developed RVO during the follow-up period and those who did not. Retinal vein occlusion included both central retinal vein occlusion and branch retinal vein occlusion. All included patients had a confirmed diagnosis of glaucoma prior to HRV testing, and controls were selected from the same clinical population during the same time frame. To enhance comparability between groups, patients with similar baseline demographics, including age and sex, were included. As this was a retrospective case-control analysis, randomization was not applicable. Patients with concurrent ocular diseases that could affect the functional and anatomical status, such as optic nerve disease, advanced age-related macular degeneration (AMD), diabetic retinopathy, high myopia with axial length ≥ 26 mm, and poor image quality, were also excluded (Fig 1).

All patients underwent complete ophthalmic evaluations, including best-corrected visual acuity, Goldmann applanation tonometry, fundoscopy, swept-source optical coherence tomography (SS-OCT) (DRI Triton, Topcon, Tokyo, Japan), and Humphrey visual field (VF) examination using the Swedish interactive threshold Standard 24−2 algorithm (Carl Zeiss Meditec, Dublin, CA, USA). Patients were also referred to the internal medicine department for HRV tests using a Medicore Heart Rate Analyzer, Model SA-3000P (Medicore, Seoul, Korea) at the time of glaucoma diagnosis, and their medical histories were thoroughly reviewed.

### Measurements

The 3 mm diameter inner ring and 6 mm diameter outer ring were segmented into superior, inferior, nasal, and temporal quadrants, according to the Early Treatment of Diabetic Retinopathy Study (ETDRS) macular map sectors [12]. Central macular thickness (CMT), ganglion cell-inner plexiform layer (GCIPL) thickness, and each sectoral retinal thickness were measured using the OCT software.

Choroidal thickness (CT) was measured using the built-in calipers of the OCT device. Subfoveal CT was determined as the distance between the outer border of the retinal pigment epithelium (RPE) and the inner border of the suprachoroidal space [13]. To minimize inter-observer variability, two retinal specialists (J.H.L. and Y.H.P.) measured CT independently, and the average value was used.

**Fig 2:**
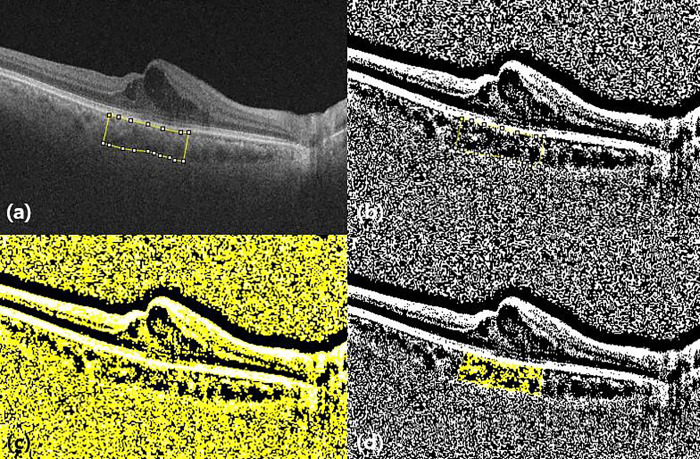
Choroidal vascularity index (CVI) measurement using Image J software. **a.** OCT image with 1,500 µm segmentation block of the subfoveal choroidal area. **b.** Image binarization using the Niblack autolocal threshold. **c.** Applying the color threshold tool, added the stromal area to the region of interest (ROI) manager. **d.** CVI was calculated as the ratio of luminal area (LA) to total choroidal area (TCA).

The choroidal vascularity index (CVI) was evaluated to assess the vascularity status of the choroid. The selected image was segmented following the protocol described by Agrawal et al., and image binarization was performed using the ImageJ 1.54 software (National Institute of Health, Bethesda, MD, USA) [14,15]. Using the polygonal selection tool, the subfoveal choroidal area with a width of 1,500 microns, demonstrating the total subfoveal circumscribed choroidal area (TCA), was selected and then added to the regions of interest (ROI) manager. The image was converted into 8-bit, and a Niblack auto local threshold was applied. After applying the color threshold tool, the stromal area (SA) was added to the ROI manager. The TCA and SA were merged using an “AND” operation within the ROI manager, creating a third area. The luminal area (LA) was calculated as the difference between the TCA and the third area. The CVI was calculated as the ratio of LA to TCA (CVI = LA/TCA × 100) (Fig 2).

### HRV assessment

Two time-domain HRV indices were retrieved: the standard deviation of all normal-to-normal R-Rs intervals (SDNN), representing the total effects of the regulation of the autonomic nervous system, and the root mean square of successive differences between normal-to-normal R-R intervals (rMSSD), which measures short-term variation and thus estimates the high-frequency variations in heart rate and is thought to be highly correlated [16–18]. Reduced SDNN values indicated an autonomic imbalance. The average SDNN and rMSSD values for Koreans in their 60s were reported as approximately 38 ms and 23 ms, respectively [16,19].

The frequency-domain analysis included the absolute and relative powers of the low frequency (LF, 0.04–0.15 Hz), high frequency (HF, 0.15–0.4 Hz), and LF/HF ratio. The LF and HF measurements represent the sympathetic and parasympathetic modulations, respectively; thus, the LF/HF ratio depicts the overall sympathovagal balance [19]. The median LF/HF ratio among Koreans in their 60s–70s was 2.91–3.61 [19].

### Statistical analyses

Statistical analyses were performed using the SPSS software (version 24.0; IBM Corp., Armonk, NY, USA). An independent t-test was performed to compare the quantitative measures between glaucoma patients with and without RVO, while Pearson’s chi-square test was used to compare the baseline demographics of the patients. A paired t-test was performed to compare the inter-eye differences in RVO patients. The Shapiro-Wilk test was used to assess the normality of the data before applying parametric tests. Logistic regression analysis was performed to assess the predictive factors associated with the development of RVO in glaucoma patients. In the logistic regression models, the dependent variable was coded as 1 for glaucoma patients without RVO (control group) and 0 for those with RVO. Variables with *P* < 0.20 in univariate analyses were initially considered for multivariable logistic regression. Given the limited number of retinal vein occlusion events, a parsimonious multivariable model including a reduced set of clinically relevant variables was constructed to minimize potential overfitting. Statistical significance was defined using an alpha level of 0.05.

## Results

A total of 63 patients diagnosed with glaucoma were included in this study, among whom 29 developed RVO during the follow-up period. Table 1 presents the baseline demographic and ophthalmic characteristics of patients. No statistically significant differences were observed between the two groups in baseline intraocular pressure and VF indices, including MD and PSD. The number of glaucoma medications was comparable between the groups, although three patients in the RVO group had undergone glaucoma surgery. However, HRV parameters showed differed significantly, with patients who developed RVO showing lower SDNN and rMSSD values compared to those without RVO (SDNN: 22.12 ± 8.27 vs. 36.71 ± 24.74, *p* = 0.002; rMSSD: 16.34 ± 9.55 vs. 29.87 ± 31.58, *p* = 0.022) (Table 1). The time from glaucoma diagnosis to RVO development varied among patients, with a mean duration of 3.36 ± 4.27 years.

**Table 1:** Baseline Characteristics and Comparisons of Glaucoma Patients According to the Development of RVO.

	Glaucoma with RVO (n = 29)	Glaucoma without RVO (n = 34)	*P* value
Age (years)	66.03 ± 9.86	64.79 ± 10.70	0.636 ^†^
Sex, male:female, n	13:16	13:21	0.596 ^†^
Medication of DM, n (%)	9 (31.0%)	5 (14.7%)	0.120 ^†^
Medication of HTN, n (%)	17 (58.6%)	14 (41.2%)	0.167 ^†^
Baseline IOP (mmHg)	14.78 ± 3.73	15.28 ± 4.24	0.484 ^†^
Number of glaucoma medication (n)	1.52 ± 0.91	1.38 ± 0.86	0.634 ^†^
Baseline MD of VF (dB)	−5.98 ± 9.05	−3.70 ± 4.70	0.114 *
Baseline PSD of VF (dB)	3.88 ± 3.78	3.98 ± 3.96	0.883 *
HR (bpm)	70.07 ± 14.17	67.91 ± 10.47	0.491 *
SDNN (ms)	22.12 ± 8.27	36.71 ± 24.74	0.002 *
rMSSD (ms)	16.34 ± 9.55	29.87 ± 31.58	0.022 *
LF/HF ratio	1.40 ± 1.39	1.59 ± 1.53	0.599 *
Follow-up period (years)	8.00 ± 5.11	9.00 ± 5.20	0.446 ^†^

p values were calculated using independent t-test (*) and Pearson’s Chi-square test (†).

Statistically significant differences between two groups (*P* < 0.05) are indicated in bold.

### Optical coherence tomography parameters

We compared OCT parameters between patients with RVO and those without vascular changes. Retinal and GCIPL thicknesses measured at the acute stage of RVO in affected eyes showed significant differences between the two groups (Table 2). RVO patients had significantly lower CVI compared to those without RVO (64.62 ± 7.38 vs. 67.49 ± 5.90, *p* = 0.045). However, no significant difference was observed in subfoveal CT (241.24 ± 81.15 vs. 258.68 ± 84.71, *p* = 0.350) (Table 2). Additionally, when comparing the structural components between the fellow eye of the RVO patients, which is the non-affected side, and non-RVO patients, no statistically significant differences were found (S1 Table).

**Table 2:** Comparison between OCT parameters in the affected eyes of patients with Retinal vein occlusion and controls.

			Glaucoma with RVO (n = 29)	Glaucoma without RVO (n = 68)	*P* value
Retinal thickness (um)		CMT	420.59 ± 207.98	232.28 ± 19.81	**<0.001**
	3 mm	Superior	398.03 ± 127.01	297.06 ± 20.45	**<0.001**
		Nasal	402.38 ± 139.07	300.50 ± 19.08	**<0.001**
		Inferior	395.07 ± 141.48	287.04 ± 23.15	**<0.001**
		Temporal	387.07 ± 135.09	282.35 ± 23.14	**<0.001**
	6 mm	Superior	325.31 ± 80.23	257.54 ± 20.81	**<0.001**
		Nasal	338.48 ± 75.87	271.56 ± 20.77	**<0.001**
		Inferior	312.72 ± 97.27	235.87 ± 19.02	**<0.001**
		Temporal	304.07 ± 71.64	237.87 ± 20.34	**<0.001**
GCIPL thickness (um)		Mean	74.02 ± 17.04	62.70 ± 9.38	**0.002**
		Center	82.72 ± 61.84	40.07 ± 8.24	**0.001**
	3 mm	Superior	86.48 ± 27.04	78.91 ± 13.90	0.162
		Nasal	88.24 ± 22.54	79.72 ± 13.26	**0.022**
		Inferior	82.03 ± 24.40	71.97 ± 17.04	**0.022**
		Temporal	78.48 ± 32.44	70.19 ± 15.41	0.198
	6 mm	Superior	65.86 ± 23.04	56.76 ± 9.48	**0.048**
		Nasal	66.90 ± 16.13	62.35 ± 7.95	0.158
		Inferior	57.17 ± 22.46	48.79 ± 8.60	0.060
		Temporal	58.31 ± 14.82	55.53 ± 8.71	0.352
Subfoveal Choroidal Thickness (um)			241.24 ± 81.15	258.68 ± 84.71	0.350
Choroidal Vascularity Index			64.62 ± 7.38	67.49 ± 5.90	**0.045**

RVO = retinal vein occlusion; GCIPL = ganglion cell-inner plexiform layer.

Statistically significant differences between two groups (*P* < 0.05) by independent t-test are indicated in bold.

#### Inter-eye measurements.

In the group analysis of RVO patients, the affected and unaffected eyes showed no functional differences in the VF test, but significant differences were observed in retinal thickness (S2 Table). Mean GCIPL thickness, as well as center and nasal GCIPL thickness within the 3 mm diameter inner ETDRS grid, differed significantly between the affected and unaffected eyes (*p* = 0.005, *p* = 0.002, *and* *p* = 0.045, respectively). The inter-eye comparison of subfoveal CT and CVI showed no statistically significant differences (CT: 221.07 ± 96.92 vs. 241.24 ± 81.15, *p* = 0.574; CVI; 65.46 ± 7.77 vs. 64.62 ± 7.38, *p* = 0.196).

### Predictive factors affecting the development of RVO

**Fig 3:**
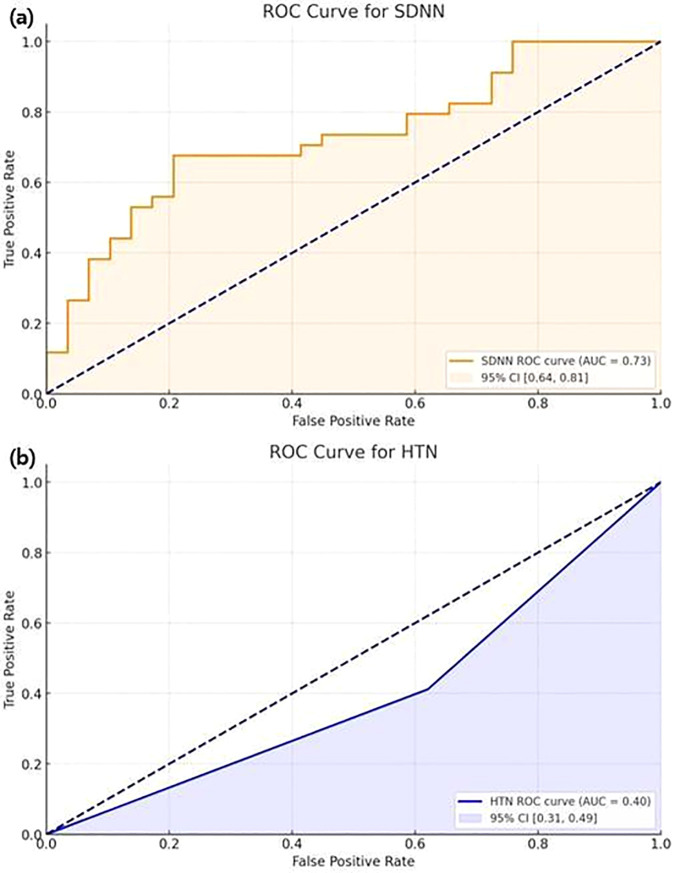
Receiver operating characteristic curve (ROC) for the prediction of retinal vein occlusion (RVO) based on the standard deviation of NN interval (SDNN) and hypertension (HTN).

Factors associated with the development of RVO in glaucoma patients were analyzed (Table 3). In univariate analysis, medication for diabetes (*p* = 0.120), systemic hypertension (*p* = 0.098), baseline MD (*p* = 0.085), subfoveal CT (*p* = 0.081), CVI (*p* = 0.049), and HRV parameters, including SDNN (*p* = 0.008) and rMSSD (*p* = 0.057) were significantly associated with RVO development. Given the limited number of RVO events, a parsimonious multivariable logistic regression model including SDNN, systemic hypertension, and baseline MD was applied. In this model, none of the variables remained statistically significant, although the direction of association for SDNN was consistent with the univariate analysis. Receiver operating characteristic (ROC) curve analysis demonstrated that SDNN showed moderate discriminatory ability for RVO occurrence, with an area under the ROC curve (AUC) of 0.73 (95% CI: 0.64–0.81), while systemic hypertension demonstrated limited discriminatory performance (AUC: 0.40, 95% CI: 0.31–0.49). (Fig 3).

**Table 3:** Factors Associated with the Development of retinal Vein Occlusion.

Variables	Univariate		Multivariate	
	OR (95% CI)	*P* value	OR (95% CI)	*P* value
Age (years)	0.988 (0.941-1.038)	0.630^†^		
Sex, male:female, n	1.313 (0.479–3.593)	0.596*		
Medication of DM	0.383 (0.112-1.314)	0.120*		
Medication of HTN	0.428 (0.155-1.180)	0.098*	0.840 (0.260-2.711)	0.771
Baseline MD of VF (dB)	1.051 (0.993-1.112)	**0.085** ^**†**^	1.001 (0.938-1.067)	0.986
Baseline PSD of VF (dB)	1.008 (0.915-1.109)	0.875^†^		
Subfoveal CT (um)	1.004 (1.000-1.008)	**0.081** ^**†**^		
CVI	291.249 (1.018-83329.762)	**0.049** ^**†**^		
HR (bpm)	0.985 (0.946-1.027)	0.485^†^		
SDNN (ms)	1.094 (1.023-1.170)	**0.008** ^**†**^	1.023 (0.979-1.069)	0.313
rMSSD (ms)	1.055 (0.998-1.114)	**0.057** ^**†**^		
LF/HF ratio	1.100 (0.776-1.560)	0.593^†^		
Follow-up period (years)	1.039 (0.942-1.146)	0.439^†^		

MD = mean deviation; PSD = pattern standard deviation; VF = visual field; CT = choroidal thickness; CVI = choroidal vascularity index; HR = heart rate; SDNN = standard deviations of NN interval; rMSSD = root-mean-square of successive differences; LF = low frequency; HF = high frequency.

*P* values were calculated using Pearson’s Chi-square test (*) and logistic regression analysis (†).

Variables with *P* < 0.20 in univariate analyses were initially considered for multivariable logistic regression. Given the limited number of retinal vein occlusion events, a parsimonious multivariable model including a reduced set of clinically relevant variables was constructed to minimize potential overfitting.

In the regression models, the outcome variable was coded as 1 for glaucoma patients without retinal vein occlusion (RVO) (control group) and 0 for those with RVO. Accordingly, odds ratios (ORs) greater than 1 indicate a higher likelihood of belonging to the control group, whereas ORs less than 1 indicate an increased likelihood of RVO.

## Discussion

In this study, we investigated potential risk factors for RVO development in glaucoma patients and found that systemic hypertension medication uses and lower HRV parameters, including SDNN and rMSSD, were significantly associated with RVO occurrence. Additionally, RVO-affected eyes exhibited lower CVI compared to glaucomatous eyes without RVO, suggesting systemic vascular involvement in RVO pathogenesis among glaucoma patients.

Previous studies have demonstrated the relationship between glaucoma and RVO development. In glaucoma patients, RVO is believed to arise from glaucomatous structural changes, such as modifications at the lamina cribrosa, or it may coexist with retinal hemodynamic instability [9,20]. While glaucoma alone can cause severe visual dysfunction, the onset of RVO can accelerate this deterioration, resulting in irreversible ophthalmic impairment. Thus, identifying risk factors and focusing on preventing RVO in glaucoma patients is critical.

Our study demonstrated that lower SDNN values, which represents dysregulation of the autonomic nervous system, were associated with the presence of RVO in glaucoma patients. Cardiovascular assessment of autonomic dysfunction via HRV is widely used because it is non-invasive and relatively easy to implement [21]. Previous research has shown that patients with low HRV had a higher risk of developing diabetic retinopathy [17,22]. Ocular blood flow is regulated by both direct and indirect mechanisms of the autonomic system [23]. Two separate vascular systems, retinal and uveal, primarily represented as choroidal vasculature, are influenced by autoregulatory influences, either indirectly or directly [23]. However, the precise impact of the autonomic nervous system on retinal vasculature remains ambiguous, highlighting the importance of systemic hemodynamic changes [24]. Autonomic dysfunction disrupts the balance of sympathetic and parasympathetic tone, leading to systemic imbalances in vasoconstriction and vasodilatation [24,25]. Our results demonstrated that patients who developed RVO had lower SDNN and rMSSD at baseline. This suggests that RVO patients may have a vulnerable autonomic nervous system, leading to an imbalance in vascular constriction and dilation. This dysfunction, similar to that seen in diabetic retinopathy, may ultimately contribute to the development of retinopathy.

Several retinal vascular diseases, including diabetic retinopathy, age-related macular degeneration, and retinopathy of prematurity, involve choroidal vascular changes that either accompany or precede retinal vascular alterations [26,27]. However, choroidal involvement in RVO remains unclear, with conflicting evidence on whether eyes affected by RVO have higher or lower CVI compared to their fellow eyes [14,26,28]. Some studies suggest that impaired retinal venous outflow in RVO could cause extracellular fluid to shift towards the choroid, leading to subsequent choroidal stromal swelling and congestion. Aribas et al. [26] reported that compared with healthy control eyes, the non-affected fellow eyes of the RVO patients had lower CVI. Our results showed no statistically significant differences in inter-eye comparison of CT and CVI values between the affected and fellow eyes of RVO patients (S2 Table). However, when comparing the CVI of RVO-affected eyes to that of the control participants, who have glaucomatous eyes without RVO, the RVO-affected eyes had significantly lower CVI (Table 2). Additionally, the fellow eyes of RVO patients tended to have lower CVI compared to the control participants (S1 Table). RVO is a condition strongly influenced by systemic vascular health. The decreased CVI observed in RVO patients compared to non-RVO patients in our study indicates alterations in choroidal vessels, which are partially associated with systemic vascular disease. Since glaucoma patients typically exhibit reduced CVI compared to healthy eyes, this may have contributed to the lack of a statistical difference in CVI between RVO-affected eyes and their non-affected fellow eyes [29].

HRV assessment is noninvasive, rapid, and relatively low-cost, suggesting potential feasibility in routine glaucoma clinics. Nevertheless, HRV should currently be regarded as an adjunctive risk stratification tool rather than a screening test, pending further prospective and cost-effectiveness studies.

This study had several limitations. First, due to the limited number of glaucoma patients who underwent the HRV test and eventually developed RVO, we could not perform a subgroup analysis based on the type of glaucoma, such as normal-tension or primary open-angle glaucoma. In addition, because of the retrospective design and limited sample size, a formal power analysis was not conducted, and the possibility of insufficient statistical power to detect small effect sizes cannot be excluded. Although multivariate regression was used to adjust for potential confounders, residual confounding cannot be entirely ruled out. Because of the limited number of RVO events, CRVO and BRVO were analyzed together, precluding subtype-specific analyses. Another limitation is that HRV results can be affected by antihypertensive drugs such as angiotensin-converting enzyme inhibitors (ACE) and ß-blockers, which could increase high-frequency HRV and reduce low- and mid-frequency bands, respectively [30]. Although we accounted for patients’ underlying metabolic diseases, detailed information regarding the specific antihypertensive medication in use — including the distinction between systemic and topical agents — was not consistently available due to the retrospective study design. Consequently, separate analysis according to systemic versus topical medication use could not be performed, and this unmeasured drug exposure may have influenced the HRV results. Finally, HRV was measured only at baseline, and autonomic function may have changed over time prior to the development of RVO. This temporal gap limits causal inference and suggests that HRV should be interpreted as a baseline risk marker rather than a dynamic predictor.

## Conclusion

HRV is a non-invasive and relatively easily accessible method for assessing autonomic dysfunction. To our knowledge, this is the first reported study to explore the association between autonomic dysfunction, reflected by reduced HRV parameters, and the development of RVO in glaucoma patients. Although the exact nature of this relationship requires further clarification, our findings suggest that reduced HRV may be associated with increased vulnerability to retinal vascular complications in glaucoma patients. Accordingly, HRV may therefore serve as a potential marker of susceptibility to RVO in glaucoma patients. Further prospective studies with larger cohorts are warranted to clarify the temporal and causal relationship between HRV and RVO development. In addition, incorporating systemic parameters such as blood pressure and cardiovascular comorbidities may improve understanding of the role of autonomic dysfunction in retinal vascular pathophysiology.

## Supporting information

S1 Table Comparison between OCT parameters in the fellow eyes of patients with retinal vein occlusion and controls. (DOCX)

S2 Table Inter-eye difference in patients with retinal vein occlusion. (DOCX)
