# Supplementary material for: Hemodynamic instability and retinal vein occlusion in glaucoma: Comparative analysis of heart rate variability and choroidal perfusion
Source: PLoS One. 2026 Mar 6;21(3):e0324110. doi: 10.1371/journal.pone.0324110 (PMC12965598; doi:10.1371/journal.pone.0324110)
Supplement: S1 Table — (DOCX) [file pone.0324110.s001.docx]

**Supporting Information 1 Table.** Comparison between OCT parameters in the fellow eyes of patients with retinal vein occlusion and controls

|  |  |  | **Glaucoma with RVO**  **(n=29)** | **Glaucoma without RVO (n=68)** | | ***P* value** | |
| --- | --- | --- | --- | --- | --- | --- | --- |
| Retinal thickness (um) |  | CMT | 240.90 ± 28.39 | 232.28 ± 19.81 | 0.145 | |  |
|  | 3 mm | Superior | 299.69 ± 34.53 | 297.06 ± 20.45 | 0.642 | |  |
|  |  | Nasal | 306.66 ± 32.51 | 300.50 ± 19.08 | 0.247 | |  |
|  |  | Inferior | 293.59 ± 39.99 | 287.04 ±23.15 | 0.415 | |  |
|  |  | Temporal | 286.48 ± 37.69 | 282.35 ± 23.14 | 0.511 | |  |
|  | 6 mm | Superior | 262.45 ± 30.57 | 257.54 ± 20.81 | 0.361 | |  |
|  |  | Nasal | 278.24 ± 34.17 | 271.56 ± 20.77 | 0.240 | |  |
|  |  | Inferior | 247.10 ± 32.37 | 235.87 ± 19.02 | 0.089 | |  |
|  |  | Temporal | 244.24 ± 30.53 | 237.87 ± 20.34 | 0.230 | |  |
| GCIPL thickness (um) |  | Mean | 62.99 ± 13.94 | 62.70 ± 9.38 | 0.904 | |  |
|  |  | Center | 41.86 ± 15.34 | 40.07 ± 8.24 | 0.557 | |  |
|  | 3 mm | Superior | 78.86 ± 18.17 | 78.91 ± 13.90 | 0.988 | |  |
|  |  | Nasal | 77.41 ± 22.52 | 79.72 ± 13.26 | 0.610 | |  |
|  |  | Inferior | 71.90 ± 24.13 | 71.97 ± 17.04 | 0.986 | |  |
|  |  | Temporal | 70.79 ± 20.05 | 70.19 ± 15.41 | 0.873 | |  |
|  | 6 mm | Superior | 56.79 ± 12.44 | 56.76 ± 9.48 | 0.990 | |  |
|  |  | Nasal | 61.79 ± 14.19 | 62.35 ± 7.95 | 0.843 | |  |
|  |  | Inferior | 52.24 ± 11.44 | 48.79 ± 8.60 | 0.106 | |  |
|  |  | Temporal | 55.03 ± 14.41 | 55.53 ± 8.71 | 0.864 | |  |
| Subfoveal Choroidal Thickness (um) | | | 221.07 ± 96.92 | 258.68 ± 84.71 | 0.058 | |  |
| Choroidal Vascularity Index | | | 65.46 ± 7.77 | 67.49 ± 5.90 | 0.162 | |  |

RVO = retinal vein occlusion; GCIPL = ganglion cell-inner plexiform layer

Independent t-test was performed for statistical analysis
