# Supplementary material for: Hemodynamic instability and retinal vein occlusion in glaucoma: Comparative analysis of heart rate variability and choroidal perfusion
Source: PLoS One. 2026 Mar 6;21(3):e0324110. doi: 10.1371/journal.pone.0324110 (PMC12965598; doi:10.1371/journal.pone.0324110)
Supplement: S2 Table — (DOCX) [file pone.0324110.s002.docx]

**Supporting Information 2 Table.** Inter-eye difference in patients with retinal vein occlusion

|  | | | **RVO eye**  **(n = 29)** | **Control eye**  **(n = 29)** | ***P* value** |
| --- | --- | --- | --- | --- | --- |
| Lens, phakia:pseuophakia | | | 25:4 | 23:6 | 0.487 ^†^ |
| Baseline IOP (mmHg) | | | 14.93 ± 4.03 | 14.62 ±3.47 | 0.607 * |
| Baseline MD of VF (dB) | | | -6.04 ± 9.30 | -5.92 ± 8.99 | 0.930 * |
| Baseline PSD of VF (dB) | | | 3.65 ± 3.22 | 4.10 ± 4.33 | 0.657 * |
| Follow up MD of VF (dB) | | | -8.83 ± 9.49 | -7.60 ± 9.39 | 0.328 * |
| Follow up PSD of VF (dB) | | | 5.53 ± 3.52 | 5.41 ± 4.10 | 0.885 * |
| CMT | | | 420.59 ± 207.98 | 240.90 ± 28.39 | **<0.001 *** |
| Retinal thickness (um) | 3 mm | Superior | 398.03 ± 127.01 | 299.69 ± 34.53 | **<0.001 *** |
|  |  | Nasal | 402.38 ± 139.07 | 306.66 ± 32.51 | **0.001 *** |
|  |  | Inferior | 395.07 ± 141.48 | 293.59 ± 39.99 | **0.001 *** |
|  |  | Temporal | 387.07 ± 135.09 | 286.48 ± 37.69 | **0.001 *** |
|  | 6 mm | Superior | 325.31 ± 80.23 | 262.45 ± 30.57 | **0.001 *** |
|  |  | Nasal | 338.48 ± 75.87 | 278.24 ± 34.17 | **<0.001 *** |
|  |  | Inferior | 312.72 ± 97.27 | 247.10 ± 32.37 | **0.001 *** |
|  |  | Temporal | 312.72 ± 97.27 | 244.24 ± 30.53 | **<0.001 *** |
| GCIPL mean | | | 74.02 ± 17.04 | 62.99 ± 13.94 | **0.005 *** |
| GCIPL center | | | 82.72 ± 61.84 | 41.86 ± 15.34 | **0.002 *** |
| GCIPL thickness (um) | 3 mm | Superior | 86.48 ± 27.04 | 78.86 ± 18.17 | 0.231 * |
|  |  | Nasal | 88.24 ± 22.54 | 77.41 ± 22.52 | **0.045 *** |
|  |  | Inferior | 82.03 ± 24.40 | 71.90 ± 24.13 | 0.090 * |
|  |  | Temporal | 78.48 ± 32.44 | 70.79 ± 20.05 | 0.228 * |
|  | 6 mm | Superior | 65.86 ± 23.04 | 56.79 ± 12.44 | 0.075 * |
|  |  | Nasal | 66.90 ± 16.13 | 61.79 ± 14.19 | 0.159 * |
|  |  | Inferior | 57.17 ± 22.46 | 52.24 ± 11.44 | 0.331 * |
|  |  | Temporal | 58.31 ± 14.82 | 55.03 ± 14.41 | 0.360 * |
| Subfoveal Choroidal Thickness (um) | | | 221.07 ± 96.92 | 241.24 ± 81.15 | 0.574 * |
| Choroidal Vascularity Index | | | 65.46 ± 7.77 | 64.62 ± 7.38 | 0.196 * |

IOP = intraocular pressure; MD = mean deviation; PSD = pattern standard deviation; CMT = central macular thickness; GCIPL = ganglion cell inner plexiform layer

p values were calculated using paired t-test (*) and Pearson’s Chi-square test (†)

Statistically significant differences between two groups (*P*<0.05) are indicated in bold
